# Treatment with Granulocyte-colony Stimulating Factor (G-CSF) is not associated with Increased Risk of Brain Metastasis in Patients with *De Novo* Stage IV Breast Cancer

**DOI:** 10.7150/jca.63159

**Published:** 2021-07-25

**Authors:** Takeo Fujii, Hasan Rehman, Su Yun Chung, Janice Shen, James Newman, Vernon Wu, Adam Hines, Elham Azimi-Nekoo, Fatima Fayyaz, Meeyoung Lee, George Raptis, Mikala Egeblad, Xinhua Zhu

**Affiliations:** 1 Division of Hematology and Medical Oncology, Northwell Health Cancer Institute, Northwell Health, Lake Success, New York, USA; 2 Cold Spring Harbor Laboratory, Cold Spring Harbor, New York, USA

**Keywords:** Breast cancer, brain metastasis, granulocyte-colony stimulating factor

## Abstract

**Background:** Survival outcome after developing brain metastasis is poor and there is an unmet need to identify factors that can promote brain metastasis. Granulocyte-colony stimulating factor** (**G-CSF) is given to support neutrophil recovery after myelosuppressive chemotherapy to some patients. However, there is emerging evidence that neutrophils can promote metastasis, including through the formation of neutrophil extracellular traps (NETs), scaffolds of chromatin with enzymes expelled from neutrophils to the extracellular space. In animal models, G-CSFs can induce NETs to promote liver and lung metastasis. The primary objective of this study was to test the association between G-CSF use and the later incidence of brain metastasis.

**Methods:** Patients with *de novo* Stage IV breast cancer, without known brain metastasis at the time of initial diagnosis, were identified from electronic medical records covering the period from 1/1/2013 to 12/31/2020 at Northwell Health. Univariate and multivariate logistic regression models were used to test the association between variables of interest, including G-CSF use, and brain metastasis.

**Results:** A total of 78 patients were included in the final analysis. Among those 78 patients, 24 patients (30.8%) had received G-CSF along with chemotherapy at least once. In logistic regression models, G-CSF use was not a significant factor to predict brain metastasis (OR 1.89 [95%CI 1.89-5.33]; P=0.23). Interestingly, in multivariate logistic models, pulmonary embolism (PE)/deep venous thrombosis (DVT) was a significant predictive factor of brain metastasis (OR 6.74 [95%CI 1.82-25.01]; P=0.004) (38.5% vs 21.5%).

**Conclusions:** The use of G-CSF was not associated with increased risk of brain metastasis in patients with *de novo* Stage IV breast cancer. Interestingly, PE/DVT, which can be associated with elevated NETs, was associated with brain metastasis. Further studies are warranted to determine whether DVT/PE with or without elevated NETs levels in the blood, is predictive of developing brain metastasis in patients with *de novo* Stage IV breast cancer.

## Introduction

In the United States, breast cancer is the most common newly diagnosed non-cutaneous cancer and the second most common cause of cancer-related death in women 1. Neoadjuvant or adjuvant chemotherapy, including with anthracycline based regimens and taxane, along with surgery and radiation therapy is the current standard of care for localized breast cancers 2.

Granulocyte colony stimulating factor (G-CSF) has been recommended by the National Comprehensive Cancer Network (NCCN) 2 for dose dense regimens with a high likelihood of causing treatment-related neutropenia, such as the above mentioned anthracycline based regiments. Filgrastim (Neupogen®) and pegfilgrastim (Neulasta®) are manufactured forms of G-CSF commonly used to stimulate the bone marrow to produce mature granulocytes as well as proliferation and differentiation of neutrophil precursors. These manufactured forms of G-CSF are given to support the recovery from myelosuppression after cytotoxic chemotherapies or to treat febrile neutropenia. A randomized trial showed that prophylactic G-CSF use significantly decreased the risk of febrile neutropenia (1% vs. 17%, G-CSF vs. placebo) and febrile neutropenia-related hospitalization (1% vs 14%, G-CSF vs. placebo) in patients with breast cancer 3.

G-CSF clearly has clinical benefits in the short-term, but the long-term consequences of G-CSF treatment are less clear. A clinical study showed that high expression levels of endogenous G-CSF in triple-negative breast cancer are associated with poor overall survival 4. In the 4T1 breast cancer mouse model, G-CSG blockage significantly reduced lung metastasis^5^. Several studies have also documented a direct link between G-CSF and formation of neutrophil extracellular traps (NETs; scaffolds of chromatin with enzymes expelled from neutrophils to the extracellular space) 5, 6. When treating healthy mice with recombinant human G-CSF, an increase in neutrophils and platelet-activating factor stimulation resulted in dose-dependent increase in NETs in blood 7. In the same study, treating 4T1 breast cancer bearing mice with an G-CSF neutralizing antibody prevented accumulation of neutrophils and reduced their sensitization toward NET formation *in vitro* 7.

NETs were originally identified as a mechanisms of microorganisms killing 8, 9. Several studies using murine models of breast cancer have documented that NETs promote metastasis to liver or lung 10, either by physically trapping circulating tumor cells or by promotion the exit from a quiescent state through extracellular matrix remodeling that turn on integrin signaling in the cancer cells 5, 11. NETs can also promote cancer metastasis to liver by binding to a transmembrane receptor, CCDC25, on cancer cells 12 to stimulate invasion. Liver, lung, and brain are, together with bones, the most common sites of breast cancer metastasis. With regard to brain metastasis in breast cancer, a recent study demonstrated that phosholylation of EZH2, which is highly expressed in brain metastatic breast cancer cells, causes *c-JUN* upregulation and increased G-CSF secretion. This lead to increased neutrophil recruitment and increased formation of breast cancer brain metastasis, while blocking G-CSF resulted in fewer brain metastasis in multiple mouse models 13.

In non-metastatic setting, the majority of the patients are expected to receive G-CSF as a part of standard care chemotherapy regimen. Thus, it is difficult to compare long-term outcome for patients who receive chemotherapy(s) with or without G-CSF in a retrospective study. In contrast, G-CSF is not routinely used in metastatic setting. Approximately 15-30% of patients with metastatic breast cancer develop brain metastasis 14. The survival outcome after patients develop brain metastasis is poor, with a median survival after diagnosis with brain metastasis of just 6-12 months 15, 16. There is no drug approved specifically to treating brain metastasis, with the current standard of care limited to radiation and surgery. Therefore, identifying factors that may drive brain metastasis so that these can be targeted is a critical clinical need.

In addition to their ability to promote metastasis, formation of NETs has also been linked to increased risk of thrombus formation including deep venous thrombosis (DVT) and pulmonary embolism (PE) 17. A clinical study showed NETs throughout thrombi obtained from endovascular thrombectomy in all patients with ischemic stroke 18. In murine experiments, it was further shown that NET formation can occur concomitant with venous thrombi in the lung 7. Particularly regarding NETs and DVT/PE in patients with cancer, a prospective observational cohort study showed that in patients with cancer, elevated NET levels in blood are associated with high cumulative incidence of venous thromboembolism 19 and another study reported that NETs in blood is a prognostic factor in patients with terminal cancer 20. Although there are many other causes of PE/DVT than excessive NET formation, presence of PE/DVT may be a clinical indicator of a particular high activation status of NET formation. However, its association with brain metastasis is unknown.

Given the mounting evidence that G-CSF promote breast cancer metastasis in mouse models by acting on neutrophils to induce NETs as well as through other mechanisms, we hypothesized that patients who received G-CSF along with chemotherapy(s) have higher incidence of brain metastasis than those who did not receive G-CSF. The primary objective of this study was to test for a potential association between G-CSF use and the incidence of brain metastasis. The secondary objectives were to test the association between G-CSF use and brain metastasis free survival and overall survival (OS) and to test for an association between PE/DVT and brain metastasis and G-CSF use in patients with *de novo* stage IV disease without brain metastasis at the time of diagnosis.

## Methods

### Study population

This retrospective chart review study was approved by the Institutional Review Board (protocol number: 20-0879) at the Feinstein Institutes for Medical Research at Northwell Health and a waiver of informed consent was granted based on the study's retrospective nature. We reviewed the electronic medical record (EMR) covering the period from 1/1/2013 to 12/31/2020 to identify patients with *de novo* stage IV breast cancer who 1) did not have confirmed brain metastasis at the time of diagnosis, and 2) had been treated with at least one cycle of any type of chemotherapy(s) with or without G-CSF at the Northwell Health Cancer Institute. G-CSF used was determined by each treatment physician based on clinical guidelines 2, 21. We excluded patients diagnosed with co-existing or prior malignancies except for cutaneous malignancies, which had been appropriately surgically treated.

### Data collection

From the EMR, we extracted age at diagnosis, ethnicity, menopausal status at diagnosis, histological type (ductal, lobular, or others), hormone receptor (HR) status, the human epidermal growth factor 2 (HER2) receptor status, smoking status, existence of pulmonary embolism (PE) and/or deep venous thrombosis (DVT) which was defined any PE/DVT found after the initial diagnosis, hormonal therapy, HER2 targeted therapy, immunotherapy, and G-CSF use. HR positivity was defined as positivity for estrogen receptor (ER) and/or progesterone receptor (PR) by immunohistochemistry staining. ER and PR positivity was defined based on the American Society of Clinical Oncology (ASCO)/College of American Pathologists (CAP) guideline. HER2 positivity was defined as a HER2/CEP17 fluorescence in situ hybridization (FISH) ratio of ≥2.0 and/or an immunohistochemical (IHC) staining score of 3+ 22.

### Statistical analysis

Standard descriptive statistics and frequency tabulation were used to summarize data. The chi-square test and Fisher's exact test were used to evaluate the association between two categorical variables. The Kruskal-Wallis test was used to compare the distributions of continuous variables among different groups. Univariate and multivariate logistic regression models were used to investigate the association between each variable and incidence of brain metastasis. Univariate and multivariate cox proportional hazard models were used to investigate the association between each variable and brain metastasis free survival (BMFS) and OS. BMFS was defined as the time from initial diagnosis to that of brain metastasis or last follow-up. OS was defined as the time from the date initial diagnosis to that of death or last follow-up. Patients who have not experienced the event (brain metastasis or death) was censored at the time of last follow-up. Variables with p-values<0.05 in univariate analysis and G-CSF use were included in multivariate analysis. A Kaplan-Meier estimate with a log-rank test was used for BMFS. All tests were two-sided and p-values <0.05 were considered statistically significant. All analyses were carried out using STATA ver. 14 software.

## Results

### Patient Characteristics

A total of 78 patients were included in the final analysis. Among these 78 patients, 24 patients (30.8%) received G-CSF. There were no significant differences in variables of interest between patients treated with G-CSF and those who were not treated with G-CSF, except for HER2 targeted therapy: G-CSF use was associated with HER2 targeting therapy. Fourteen of 24 patients (58.3%) who were treated with G-CSF received HER2 targeting therapy and 18 of 54 patients (33.3%) who were not treated with G-CSF received it (P=0.04). Baseline patient characteristics are show in Table 1.

### The incidence of brain metastasis

A total of 23 patients (29.5%) developed brain metastasis during the follow-up period. Thirteen of 54 patients treated without G-CSF use (24.1%) and 10 of 24 patients treated with G-CSF use (41.7%), a difference was not statistically significant (P=0.28). Among 13 patients who were treated without G-CSF and developed brain metastasis, 6 patients had PE/DVT (46.1%) and among 10 patients who were treated with G-CSF and developed brain metastasis, 2 patients had PE/DVT (20%). In univariate logistic regression models, G-CSF use was not a significant predictor of brain metastasis (OR 1.89 [95%CI 1.89-5.33]; P=0.23) (Table 2). Interestingly, PE/DVT was significantly associated with brain metastasis (OR 5.83 [95%CI 1.65-20.63]; P=0.006) (38.5% of patients with DVT/PE and 21.5% of those without developed brain metastasis). Since G-CSF use is the variable of our interest, we performed a multivariate analysis including G-CSF use and DVT/PE. In multivariate analysis, PE/DVT remained significant (OR 6.74 [95%CI 1.82-25.01]; P=0.004). G-CSF was not significantly associated with brain metastasis in multivariate analysis either (OR 2.37 [95%CI 0.77-7.28]; P=0.13). G-CSF use and PE/DVT were not associated with each other (P=0.744).

### Brain metastasis free survival (BMFS) and overall survival (OS)

There was no significant difference in BMFS between patients treated with or without G-CSF (Log-rank P=0.35) (Figure S1). G-CSF was neither a significant factor in predicting BMFS in univariate (HR, 1.33 [95% CI, 0.57-3.1]; P = 0.52) or multivariate cox proportional hazard models (HR, 0.85 [95% CI, 0.32-2.22]; P = 0.74) (Table S1). However, PE/DVT was associated with short BMFS in multivariate cox proportional hazard models after adjusting for other significant variables (HR, 3.08 [95% CI, 1.1-8.6]; P=0.03).

With regard to OS, G-CSF use was not a significant variable (HR, 0.53 [95% CI, 0.21-1.31]; P=0.17) in univariate cox proportional hazard models (Table S2). HER2 positivity and HER2 target treatment were associated with short OS (HR, 0.29 [95% CI, 0.11-0.75]; P=0.01, HR, 0.3 [95% CI, 0.11-0.79]; P=0.02, respectively) in univariate cox proportional hazard models, but neither variable was significant in multivariate cox proportional hazard models.

## Discussion

In this retrospective study, we found no association between G-CSF use and the development of brain metastasis in patients with newly diagnosed *de novo* stage IV breast cancer. Interestingly, PE/DVT having occurred during follow-up period was associated with high incidence of brain metastasis.

In the metastatic setting, G-CSF is currently used to support neutrophil recovery after myelosuppressive cytotoxic chemotherapies, prophylactic to maintain dose density or to treat febrile neutropenia 2. A previous meta-analysis showed that length of hospitalization (HR 0.63 [95% CI 0.49 -0.82]; P=0.0006) and time to neutrophil recovery (HR 0.32 [95% CI 0.23-0.46]; P< 0.00001) were both shorter in patients who were treated with G-CSFs than those who were not 23. These are clear clinical benefits from giving G-CSF together with cytotoxic chemotherapies, but there are previous studies raising concerns that G-CSF use may promote cancer progression, and particularly metastasis, in the context of induced neutrocytosis 24, 25. It has been shown that G‐CSF is expressed higher in triple negative breast cancer (TNBC) tumors compared to other subtypes (*P* < 0.001) 4, 26. Furthermore, high G‐CSF levels in TNBC tumors are associated with short overall survival 4, 25, 27.

Recently, it has been shown that one mean by which neutrophils can promote cancer progression is through the formation of NETs. This is of particular interest because G-CSF can induce neutrophils to form NETs 5 and because G-CSF promotes brain metastasis in a breast cancer mouse model 13.

Our current study, including 78 patients, showed that patients who received G-CSF demonstrated numerically high incidence of brain metastasis compared to those who did not (24.1% versus 41.7%), but the difference was not statistically significant (P=0.28). This result remained similar in logistic regression models (OR 1.89 [95%CI 1.89-5.33]; P=0.23). A recent paper analyzing a total of 468 patients showed that the levels of NETs in brain metastases were not significantly increased over the levels in the primary breast tumors and furthermore, the serum levels of NETs were not significantly elevated in patients with brain metastasis compared to patients without any type of metastasis 12. Together, these data suggest that G-CSF induced NETs are not likely to play a major role in promotion as drivers of metastasis. However, among the variables of interest, PE/DVT was strongly associated with brain metastasis (OR 5.83 [95%CI 1.65-20.63]; P=0.006). The association between NET and thrombosis is well established 28, 29. This result suggests that although G-CSF-induced NETs are likely to directly linked to brained metastasis, the presence of PE/DVT potentially triggered by NET, may be a surrogate predictive marker of future brain metastasis and this needs to be confirmed by further prospective studies, and if confirmed, studies to address the mechanism linking G-CSF use complicated with PE/DVT with brain metastasis need to be planned.

There are several limitations in this study. First, this is a retrospective study. Second, it was necessary to focus on *de novo* stage IV breast cancer because earlier stage breast cancer almost always utilize G-CSF in neoadjuvant or adjuvant therapy but this may not represent the pattern of metastasis in other settings of patients with breast cancer. Third, sample size is small which is partially because our patients at Northwell system are taking annual screening regularly and tend to be diagnosed with earlier stage. Due to the good compliance with annual screening, the number of patients with *de novo* stage IV was relatively small and brain metastasis, our event of interest, is relatively rare. In our study, the total sample size is 78, with 13 of 54 having not been treated with G-CSF (24.1%) and 10 of 24 treated with G-CSF (41.7%) developed brain metastasis. With our sample size, the power to detect the 17.6% difference (41.7% vs 24.1%) using α=0.05 is 0.65. With α=0.05 and β=0.8, calculated sample sizes are 150, 79, and 47, respectively to detect 15% difference (40% vs 25%), 20% difference (40% vs 20%), or 25% (40% vs 15%). Based on these calculations, we cannot exclude that G-CSF use causes an increase in brain metastasis incidence, up to about a 25% difference between treated and untreated.

In conclusion, with the noted limitations, it is encouraging that the use of G-CSF was not associated with brain metastasis in patients with *de novo* Stage IV breast cancer, although a larger study is necessary to exclude an association. Interestingly, PE/DVT, which can be associated with elevated NETs, was associated with brain metastasis and further studies are warranted to determine more directly whether the presence of NETs in blood and DVT/PE are predictive of developing brain metastasis in patients with *de novo* Stage IV breast cancer.

## Supplementary Material

Supplementary figure and tables.

## Figures and Tables

**Table 1:** Baseline patient characteristics

		G-CSF (%) (N=24)		No G-CSF (%) (N=54)		P-values
						
**Age (range, median)**		29-76 (56)		29-93 (56)		0.87
**Gender**						
** Female**		24 (100)		51 (94.4)		0.55
** Male**		0 (0)		3 (5.6)		
**Ethnicity**						
** Caucasian**		12 (50)		34 (63)		0.35
** African American**		4 (16.7)		12 (22.2)		
** Others**		6 (25)		7 (13)		
** Unknown**		2 (8.3)		1 (1.9)		
**Menopausal status**						
** Premenopause**		9 (37.5)		24 (44.4)		0.4
** Postmenopause**		15 (62.5)		26 (48.1)		
** Unknown**		0 (0)		4 (7.4)		
**Smoking Status**						
** Yes**		6 (25)		17 (31.5)		0.53
** No**		18 (75)		36 (66.7)		
** Unknown**		0 (0)		1 (1.9)		
**Active PE/DVT**						
** Yes**		3 (12.5)		10 (18.5)		0.74
** No**		21 (87.5)		44 (81.5)		
**Hormonal Status**						
** Positive**		16 (66.7)		43 (79.6)		0.22
** Negative**		8 (33.3)		11 (20.4)		
**HER2 status**						
** Positive**		14 (58.3)		20 (37)		0.08
** Negative**		10 (41.7)		34 (63)		
**Histology**						
** Ductal**		18 (75)		39 (72.2)		0.75
** Lobular**		3 (12.5)		4 (7.4)		
** Others**		2 (8.3)		7 (13)		
** Unknown**		1 (4.2)		4 (7.4)		
**Hormone treatment**						
** Yes**		15 (62.5)		40 (74.1)		0.3
** No**		9 (37.5)		14 (25.9)		
**HER2 treatment**						
** Yes**		14 (58.3)		18 (33.3)		0.04
** No**		10 (41.7)		36 (66.7)		
**Immunotherapy**						
** Yes**		6 (25)		6 (11.1)		0.17
** No**		18 (75)		48 (88.9)		

**Table 2:** Logistic regression model in incidence of brain metastasis

		Univariate analysis				Multivariate analysis			
		Odds Ratio (95%CI)		P-Values		Odds Ratio (95%CI)		P-Values	
**Age (range, median)**		0.97 (0.94-1.01)		0.16					
**Ethnicity**									
** Caucasian**		Ref							
** African American**		1.45 (0.41-5.08)		0.57					
** Others**		1.99 (0.54-7.35)		0.3					
**Menopausal status**									
** Premenopause**		Ref							
** Postmenopause**		0.49 (0.17-1.37)		0.18					
**Smoking Status**									
** No**		Ref							
** Yes**		0.84 (0.28-2.52)		0.75					
**Active PE/DVT**									
** No**		Ref				Ref			
** Yes**		5.83 (1.65-20.63)		0.006		6.74 (1.82-25.01)		0.004	
**Hormonal Status**									
** Negative**		Ref							
** Positive**		2.53 (0.66-9.76)		0.18					
**HER2 status**									
** Negative**		Ref							
** Positive**		0.86 (0.32-2.33)		0.77					
**Histology**									
** Ductal**		Ref							
** Lobular**		1.92 (0.39-9.56)		0.43					
** Others**		0.73 (0.14-3.9)		0.72					
**Hormone treatment**									
** No**		Ref							
** Yes**		1.61 (0.51-5.51)		0.41					
**HER2 treatment**									
** No**		Ref							
** Yes**		1.07 (0.39-2.92)		0.9					
**Immunotherapy**									
** No**		Ref							
** Yes**		1.33 (0.36-4.98)		0.67					
**G-CSF use**									
** No**		Ref				Ref			
** Yes**		1.89 (0.67-5.33)		0.23		2.37 (0.77-7.28)		0.13	
