# Supplementary material for: Treatment with Granulocyte-colony Stimulating Factor (G-CSF) is not associated with Increased Risk of Brain Metastasis in Patients with De Novo Stage IV Breast Cancer
Source: J Cancer. 2021 Jul 25;12(18):5687–92. doi: 10.7150/jca.63159 (PMC8364654; doi:10.7150/jca.63159)
Supplement: Supplementary file 1 — Supplementary figure and tables. [file jcav12p5687s1.pdf]

Figure S1

Kaplan-Meier curve with log-rank test in brain metastasis free survival (BMFS)

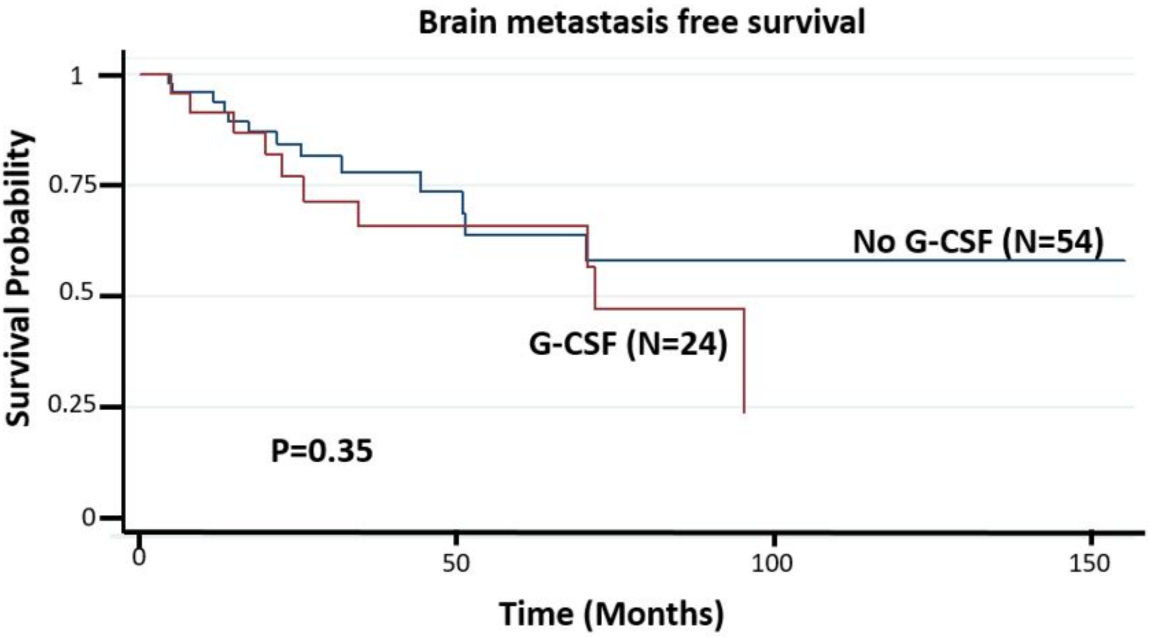

Figure S1. Kaplan-Meier curve with log-rank test in brain metastasis free survival (BMFS)

Table S1. Cox proportional hazard models in brain metastasis free survival

	Univariate analysis		Multivariate analysis	
	**Hazard Ratio (95%CI)**	**P-Values**	**Hazard Ratio (95%CI)**	**P-Values**
**Age (range, median)**	0.98 (0.94-1.01)	0.17		
**Ethnicity**				
**Caucasian**	Ref		Ref	
**African American**	3.36 (1.12-10.06)	0.03	2.65 (0.84-8.36)	0.1
**Others**	2.07 (0.72-5.96)	0.18	2.86 (0.87-9.37)	0.08
**Menopausal status**				
**Premenopause**	Ref			
**Postmenopause**	0.54 (0.23-1.28)	0.16		
**Smoking Status**				
**No**	Ref			
**Yes**	0.96 (0.37-2.45)	0.93		
**Active PE/DVT**				
**No**	Ref		Ref	
**Yes**	2.98 (1.23-7.17)	0.02	3.08 (1.1-8.6)	0.03
**Hormonal Status**				
**Negative**	Ref			

**Positive**	1.42 (0.42-4.81)	0.57		
**HER2 status**				
**Negative**	Ref			
**Positive**	0.96 (0.41-2.24)	0.92		
**Histology**				
**Ductal**	Ref			
**Lobular**	1.62 (1.47-5.56)	0.45		
**Others**	0.63 (0.14-2.74)	0.54		
**Hormone treatment**				
**No**	Ref			
**Yes**	0.72 (0.26-1.98)	0.53		
**HER2 treatment**				
**No**	Ref			
**Yes**	1.24 (0.54-2.87)	0.62		
**Immunotherapy**				
**No**	Ref			
**Yes**	1.43 (0.48-4.25)	0.53		
**G-CSF use**				
**No**	Ref		Ref	
**Yes**	1.33 (0.57-3.1)	0.52	0.85 (0.32-2.22)	0.74

Table S2. Cox proportional hazard models in overall survival

	Univariate analysis		Multivariate analysis	
	Odds Ratio (95%CI)	P-Values	Odds Ratio (95%CI)	P-Values
**Age (range, median)**	1.01 (0.98-1.04)	0.5		
**Ethnicity**				
**Caucasian**	Ref		Ref	
**African American**	3.94 (1.39-11.11)	0.01	4.78 (1.62-14.1)	0.005
**Others**	1.59 (0.57-4.47)	0.38	2.85 (0.82-9.82)	0.1
**Menopausal status**				
**Premenopause**	Ref			
**Postmenopause**	0.96 (0.45-2.06)	0.92		
**Smoking Status**				
**No**	Ref			
**Yes**	1.12 (0.51-2.5)	0.77		
**Active PE/DVT**				
**No**	Ref			
**Yes**	2.16 (0.93-5.06)	0.08		
**Hormonal Status**				
**Negative**	Ref			
**Positive**	0.52 (0.21-1.25)	0.14		
**HER2 status**				
**Negative**	Ref		Ref	
**Positive**	0.29 (0.11-0.75)	0.01	0.66 (0.09-4.96)	0.68
**Histology**				
**Ductal**	Ref			
**Lobular**	1.34 (0.39-4.62)	0.64		
**Others**	1.44 (0.53-3.9)	0.48		
**Hormone treatment**				
**No**	Ref			
**Yes**	0.79 (0.29-2.13)	0.64		
**HER2 treatment**				
**No**	Ref		Ref	
**Yes**	0.3 (0.11-0.79)	0.02	0.46 (0.06-3.46)	0.45
**Immunotherapy**				
**No**	Ref			
**Yes**	0.93 (0.28-3.15)	0.91		
**G-CSF use**				
**No**	Ref		Ref	
**Yes**	0.53 (0.21-1.31)	0.17	0.36 (0.11-1.14)	0.08
